# Additive Manufacturing of Bead-Chain-Shaped Scaffolds with AI-Based Process Optimization

**DOI:** 10.3390/polym17222973

**Published:** 2025-11-07

**Authors:** JinA Kim, Hyung Woo Kim, Young-Sam Cho

**Affiliations:** 1 Department of Mechanical Engineering, College of Engineering, Wonkwang University, 460 Iksandae-ro, Iksan 54538, Jeonbuk, Republic of Korea; kja4756@wku.ac.kr; 2 Division of Mechanical Engineering, College of Engineering, Wonkwang University, 460 Iksandae-ro, Iksan 54538, Jeonbuk, Republic of Korea; 3 MECHABIO Group, Wonkwang University, 460 Iksandae-ro, Iksan 54538, Jeonbuk, Republic of Korea; 4 Advanced Bio-Convergence Research Center, Wonkwang University, 460 Iksandae-ro, Iksan 54538, Jeonbuk, Republic of Korea

**Keywords:** scaffold, artificial intelligence, Bead-Chain-Shaped, compressive stiffness, contact area

## Abstract

Scaffolds are widely recognized as implantable alternatives in the field of tissue engineering. Among various scaffold structures, grid structures are commonly used due to their simple design and ease of fabrication. However, grid structures have a critical demerit of low mechanical stiffness compared to its own mechanical property (used material’s compressive stiffness), as the limited contact area between strands prevents effective load distribution. Several structural designs, such as triply periodic minimal surface (TPMS), modified honeycomb, and Kagome structures, have been proposed to improve compressive stiffness. Despite their mechanical advantages, these structures are limited by complex design and manufacturing processes. In this study, we propose a Bead-Chain-Shaped (BCS) scaffold, which maintains the simplicity of grid structures while enhancing compressive stiffness through the printing process alone. To optimize the printing process and enhance fabrication efficiency, we developed an artificial intelligence (AI)-based process optimization model that correlates printing parameters (pressure, printing speed, and delay time) with the resulting geometric accuracy while maintaining the designed geometry, and predicts the optimal printing conditions for the predesigned Bead-Chain-shaped (BCS) geometry. The model was then used to extract these optimal printing conditions, enabling precise dimensional control and improving overall fabrication accuracy of the Bead-Chain-Shaped (BCS) scaffold dimensions. Under the optimized printing conditions, the BCS scaffolds achieved compressive stiffness values of 61.8, 75.9, and 91.6 MPa for BCS 5545, 6040, and 6535, respectively, corresponding to increases of 11.9%, 37.3, and 65.7% compared to the control scaffold (55.3 MPa). Numerical analysis confirmed that compressive stiffness increases as strand-to-strand contact area increases. Furthermore, in vitro cell proliferation assays demonstrated no significant difference in cell proliferation compared to conventional structures (grid-structure scaffold), indicating that the proposed design does not inhibit cellular growth. These results highlight the potential of the proposed Bead-Chain-Shaped (BCS) scaffold as a promising candidate for bone tissue engineering, offering both enhanced mechanical stiffness and fabrication efficiency.

## 1. Introduction

Bone defects can result from various causes such as aging, trauma, and disease. Conventional treatments include autografts, allografts, and xenografts; however, these methods are limited by immune rejection, infection risk, donor scarcity, and limited tissue availability [1,2,3,4,5]. To address these challenges, recent advances in tissue engineering have focused on scaffolds made from U.S. Food and Drug Administration (FDA)-approved, biodegradable, and biocompatible polymeric materials [5,6,7]. Representative examples include poly(lactic acid) (PLA), poly(glycolic acid) (PGA), poly(lactic-co-glycolide) (PLGA) and poly(ε-caprolactone) (PCL) [1,4,6,8], which are widely recognized for their applications in scaffold fabrication as alternatives to traditional therapies.

An ideal scaffold design requires critical morphological parameters, including porosity, pore size, and pore interconnectivity [9,10,11,12]. These parameters not only influence bone regeneration but also facilitate oxygen and nutrient transport, cell adhesion, proliferation, migration, and vascularization [3,12]. Additionally, scaffolds must exhibit sufficient mechanical properties to withstand external loads and in vivo stresses, which are essential for long-term tissue regeneration [12,13,14].

Recently, scaffolds have been primarily fabricated using additive manufacturing (AM) technology, which offers advantages such as precise three-dimensional structures and patient-specific designs. Among various scaffold types, grid-structure scaffolds are widely used due to their simple geometry and ease of fabrication. However, conventional grid-structure scaffolds suffer from low mechanical stiffness and poor load-bearing capacity [12,15,16]. Studies have explored various alternative scaffold architectures, including triply periodic minimal surface (TRMS), Kagome, and modified honeycomb structures, to address the mechanical limitations of conventional grid scaffolds [17,18,19,20,21]. These designs have demonstrated improved mechanical stiffness and load-bearing capacity. However, despite their advantages, they often require complex mathematical modeling, leading to difficulties in both design and manufacturing processes.

**Scheme 1:**
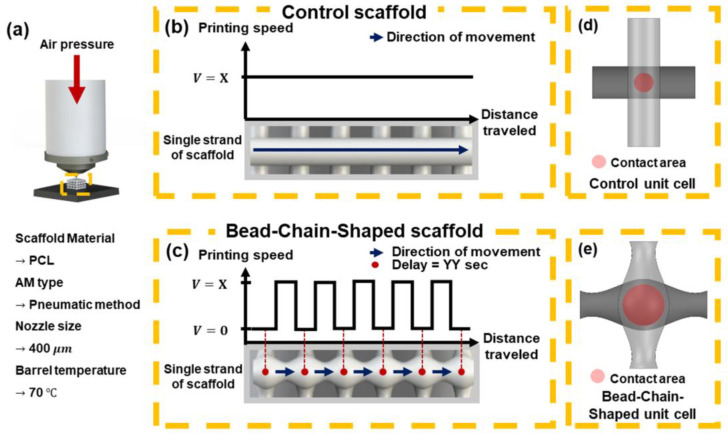
(**a**) 3D printing conditions, (**b**) printing speed of the control scaffold, (**c**) printing speed of the Bead-Chain-Shaped scaffold, (**d**) contact area image of the control scaffold, and (**e**) contact area image of the Bead-Chain-Shaped scaffold.

To overcome these limitations, this study proposes a novel scaffold design using additive manufacturing (AM) technology. The proposed scaffold, referred to as the Bead-Chain-shaped (BCS) scaffold, maintain the geometric simplicity and fabrication efficiency of conventional grid-structure scaffolds while significantly enhancing mechanical performance. As shown in Scheme 1, the Control scaffold with a conventional grid structure is printed with a constant nozzle movement speed. In contrast, the proposed BCS scaffold is fabricated through an intermittent nozzle movement pattern, involving repeated stops and moves. This stopping mechanism increases interlayer contact area, as illustrated in Scheme 1d,e, which distributes the load more evenly and enhances compressive stiffness while minimizing design and manufacturing complexity.

In recent years, numerous studies have employed artificial intelligence (AI) to improve design optimization, process control, and quality prediction in additive manufacturing (AM). Previous research has mainly focused on incorporating conventional process parameters such as temperature, laser power, and printing speed into predictive models to analyze their correlations with mechanical and geometric outcomes, thereby enhancing printing precision [22,23,24].

In this study, in addition to commonly used parameters such as pressure and printing speed, a new process variable termed delay time was introduced. By integrating this variable, the conventional grid type printing process was modified into an intermittent nozzle movement pattern, enabling the fabrication of a Bead-Chain-Shaped (BCS) scaffold. The introduction of this new variable altered the process behavior, thus necessitating a new form of process level optimization to ensure precise realization of the designed geometry. To address this, an AI-based process optimization model was developed. The model used pressure, printing speed and delay times input parameters and learned the corresponding output dimensions (D_1_ and D_2_) to predict the nonlinear relationships between process parameters and geometric outcomes. Through this model, the optimal printing conditions were determined, enabling highly accurate dimensional reproduction of the predesigned geometry and improving overall fabrication accuracy and reproducibility.

Through the optimized processing conditions with AI model, BCS scaffolds were fabricated via 3D printing and subsequently tested for compressive stiffness using a universal testing machine (UTM), and validated through numerical analysis. Additionally, reconstruction techniques were applied to compare the designed and fabricated scaffolds, followed by further numerical analysis to reassess compressive stiffness. Finally, in vitro experiments were performed to evaluate the bone regeneration potential of the fabricated scaffolds.

This study highlights that the proposed scaffold design effectively overcomes the limitations of conventional grid structures by offering enhanced mechanical properties and fabrication efficiency, achieved through and AI-based process optimization approach that refines printing parameters to improve both dimensional accuracy and production efficiency.

## 2. Materials and Methods

### 2.1. Primary Design of Scaffold

**Figure 1:**
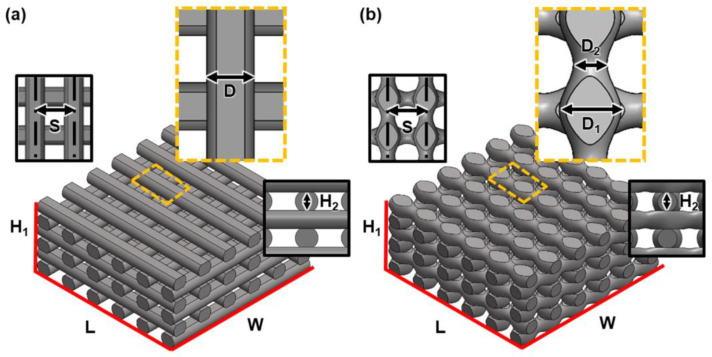
Scaffold design: (**a**) primary designed 3D grid structure model and unit cell and (**b**) primary designed 3D Bead-Chain-Shaped structure model and unit cell.

The scaffold model was designed using the 3D modeling software SolidWorks (Version 2020, Dassault Systems SolidWorks Corp., Waltham, MA, USA). The Control grid-structure scaffold was designed with dimensions of 5.5 mm in length (L), 5.5 mm in width (W), 2.4 mm in height (H_1_), 0.4 mm in single strand height (H_2_) and 1.0 mm in pitch between strands (S) as shown in Figure 1a. Strand size, D was set to 0.5 mm to achieve a porosity of 50%. The BCS scaffold, serving as the experimental group, was designed with dimensions 5.5 mm in length (L), 5.5 mm in width (W), 2.4 mm in height (H_1_), and 0.4 mm in single strand height (H_2_) as shown in Figure 1b. However, according to the values of D_1_ (a parameter related to the contact area), D_2_ and S were varied while maintaining a porosity of 50%. The common parameters of the Control grid-structure scaffold and BCS scaffold, along with the specific D_1_, D_2_ and S, values for the BCS scaffold are provided in Table 1. In addition, the Bead-Chain-Shaped scaffold is denoted as BCS for clarity. A naming convention was adopted based on the values of D_1_ and D_2_, resulting in scaffold labels such as “BCS 5545,” “BCS 6040,” and “BCS 6535” for intuitive identification.

### 2.2. Artificial Intelligence (AI) Model Architecture, Training, and Inverse Prediction

A total of 123 experimental data points, consisting of three process parameters (pressure, printing speed, and delay time) and corresponding output diameters (D_1_ and D_2_) were used to develop a predictive model. The three process parameters were employed as input variables, while the measured diameters D_1_ and D_2_ served as the output variables. Prior to training, both input and output variables were normalized using the MinMaxScaler to ensure balanced learning across variables with different scales and units.

A Multilayer Perceptron (MLP)-based Artificial Neural Network (ANN) architecture was employed to model the nonlinear relationships between the printing parameters and target diameters. To identify the optimal model configuration, Bayesian-based hyperparameter optimization was conducted using Keras Tuner Hyperband [25]. The search space included the number of neurons per layer (16–128), number of layers (1–3), dropout rate [26] (0.1–0.5), learning rate (10^−5^–10^−2^), and activation function [27] (‘ReLU’ or ‘tanh’). The best-performing configuration consisted of two hidden layers with 64 neurons per later, a dropout rate of 0.3, a learning rate of 1 × 10^−3^, and the ReLU activation function, achieving the lowest validation loss among the explored configurations. The final ANN was trained using these optimized hyperparameters with the Adam optimizer [28], which adaptively adjusts the learning rate to enhance convergence stability and training efficiency.

To ensure generalization, five-fold cross-validation was performed. The mean squared error (MSE) served as the primary loss function, and the root mean squared error (RMSE) was used for intuitive performance interpretation. The average validation MSE and RMSE across all folds were reported as final indicators.

The trained ANN was subsequently used for inverse prediction. A grid search of input parameters (pressure, printing speed, and delay time) was conducted, and corresponding D_1_ and D_2_ values were predicted. By matching the predicted and target diameters (D_1_ and D_2_), optimal process conditions were identified, enabling efficient selection of parameters that achieve desired scaffold geometries.

### 2.3. Fabrication of Bead-Chain-Shaped (BCS) Scaffold

To facilitate scaffold printing, G-code was manually generated using NC Viewer (Version 1.2.0, Toolpath Labs, Inc., Bedford Heights, OH, USA). Polycaprolactone (PCL) (MW = 37,000; Polysciences Inc., Warrington, PA, USA) pellets were loaded into the barrel of an additive manufacturing (AM) printer and melted at 70 °C for 1 h. Scaffolds were fabricated using a precision nozzle with an inner diameter of 400 μm. The “Control” scaffold was fabricated under pre-determined conditions (pressure: 180 kPa, delay time: 0 s) without applying any AI-based process optimization. In contrast, the “BCS 5545,” “BCS 6040,” and “BCS 6535” scaffolds were fabricated using optimal printing conditions derived from an AI-based prediction model. This model was trained to predict suitable combinations of pressure, printing speed, and delay time based on the target dimensions D_1_ and D_2_ enabling data driven process optimization for scaffold fabrication.

### 2.4. Analysis of Scaffold Characteristics

To evaluate the similarity between the fabricated scaffold and the designed scaffold, we first measured the porosity of the fabricated scaffold. The porosity was determined using the volume, density and weight of the scaffold. The porosity was calculated using Equation (1).

(1) $$Porosity(\%)=\frac{V_{0}-\frac{m_{s}}{\rho_{PCL}}}{V_{0}}\times 100(\%)$$

where *V*_0_ is the outer volume of the fabricated scaffold, *m_s_* is the weight of the fabricated scaffold, and $\rho_{PCL}$ is the density of PCL used during fabrication. For each scaffold type, five samples were measured.

To evaluate the characteristics of the fabricated scaffolds, including pore size and the H_2_, D, D_1_, D_2_, and S values described in Figure 1 and Table 1, we performed measurements using an optical microscope (Leica DMS1000, Leica Microsystems, Wetzlar, Germany) and a scanning electron microscope (SEM, SU380, Hitachi, Tokyo, Japan). H_2_ was examined using the optical microscope, while pore size, D, D_1_, D_2_, S were analyzed using SEM. Image analysis was performed using ImageJ (Version 1.53q, National Institutes of Health, Bethesda, MD, USA). The pore size for the top pores were measured as the average value of their horizontal pore width and vertical pore width and the side pores were measured as the average value of their pore width and pore height, respectively. H_2_ was measured as the single strand height in the non-overlapping region between strands. D was measured as strand’s width at random position in top-view SEM images while D_1_, and D_2_ were measured as the longest and shortest strand’s width in top-view SEM images, respectively. S was measured as the strand center to center distance. To evaluate the accuracy and consistency of the fabricated scaffolds, error rate (%) and precision rate (%) were calculated as dimensionless values. Error rate (%) represents the relative difference between measured mean value and the designed target value and was calculated using Equation (2), while precision rate (%) represents the relative standard deviation with respect to the measured mean value and was calculated using Equation (3). For each scaffold type, three samples were measured, and ten measurement points were used at each sample.

(2) $$Error\;rate(\%)=\left\|\frac{Measured\;mean\;value-Designed\;target\;value}{Designed\;target\;value}\right\|\times 100(\%)$$

(3) $$Precision\;rate(\%)=\left\|\frac{Standard\;deviation\;of\;measured\;value}{Measured\;mean\;value}\right\|\times 100(\%)$$

### 2.5. Reconstructing a Model Based on Fabricated Scaffold Images

To compare the differences between the designed scaffold and the fabricated scaffold, re-design was performed. In the re-design process, images of the fabricated scaffold were captured using an optical microscope. The selected images corresponded to samples with measured variable values closest to the measured average values. These images were then imported into SolidWorks, where 2D sketches were created based on the contours of the images. These sketches were subsequently used to model the 3D shape, ensuring that the final structure closely resembled the actual fabricated scaffold. The primary designed scaffold refers to the initially designed scaffold, while the secondary re-designed scaffold denotes the scaffold that was re-designed based on images of the fabricated scaffold.

### 2.6. Assessment of the Stiffness by Numerical Analysis

The mechanical compressive of the Control scaffold and BCS scaffold was predicted using ABAQUS 6.14 FEM software (Dassault Systèmes, Vélizy-Villacoublay, Île-de-France, France). The 3D models were created in SolidWorks, exported as step files, and imported into ABAQUS for simulation. The primary designed scaffold, and secondary re-designed scaffold were all analyzed under the same numerical simulation conditions. The material properties were assigned based on the characteristics of the fabricated PCL, with an elastic modulus of 400 MPa and Poisson’s ratio of 0.38. Quadratic tetrahedral elements (C3D10) were employed in the ABAQUS simulations to enhance the accuracy of the mechanical behavior representation of the scaffold. The boundary conditions were defined considering the complex curved surfaces of the scaffold design (Figure S1). At the bottom surface, the Z-direction displacement was fixed at zero along a line parallel to the X-axis and the X-direction displacement was fixed at zero along a line parallel to the Z-axis. In the Y-direction, the bottom surface was fixed with zero displacement, while the top surface was assigned a displacement of −0.32 mm, which corresponds to 10% of the scaffold height. For Von Mises stress analysis, the bottom surface was constrained under the same conditions, and a distributed load of 1 N was applied to the top surface in the Y-direction.

### 2.7. Comparison of In Vitro Cell Proliferation

Mouse calvarial preosteoblastic cells (MC3T3-E1 cells, ATCC, Manassas, VA, USA) were cultured in Dulbecco’s modified Eagle’s medium (DMEM, Gibco, New York, NY, USA) supplemented with 5% fetal bovine serum (FBS, Gibco) and 1% Antibiotic-Antimycotic 100X (Gibco) under 37 °C and 5% CO_2_ conditions in an incubator. The culture medium was exchanged every two days. Before cell seeding, the scaffolds were sterilized through a multi-step process to ensure aseptic conditions. Each scaffold was immersed in 70% ethanol and ultrasonicated for 10 min, and this step was repeated three times using freshly prepared ethanol. Afterward, the scaffolds were rinsed several times with sterile distilled water to remove residual ethanol and then exposed to ultraviolet (UV) light for 24 h under a clean bench. For each scaffold type, three samples were used at each time point (days 1, 7, and 14). MC3T3-E1 cells were seeded onto sterilized scaffolds at a density of 1 × 10^5^ cells/scaffold, with three samples per group. To assess cell proliferation within the scaffold, the Cell Counting Kit-8 (CCK-8, Dojindo Molecular Technologies, Kumamoto, Japan) assay was performed on days 1, 7, and 14. The CCK-8 solution was added to each well and incubated at 37 °C with 5% CO_2_ for 3 h. The absorbance of viable cells was then measured at 450 nm using a microplate reader (Bio Tek 800TS, Agilent, Santa Clara, CA, USA) to evaluate cell proliferation.

### 2.8. Statistical Analysis

The experimental data are presented as mean ± standard deviation. Statistical analyses were performed using GraphPad Prism 10 (GraphPad Inc, San Diego, CA, USA). A one-sample *t*-test was performed to compare the measured values of the fabricated scaffold to the designed values, and a one-way analysis of variance (ANOVA) was used for comparisons among multiple groups.

## 3. Results and Discussion

### 3.1. AI Training for Controlling Diameters of BCS Scaffolds

**Figure 2:**
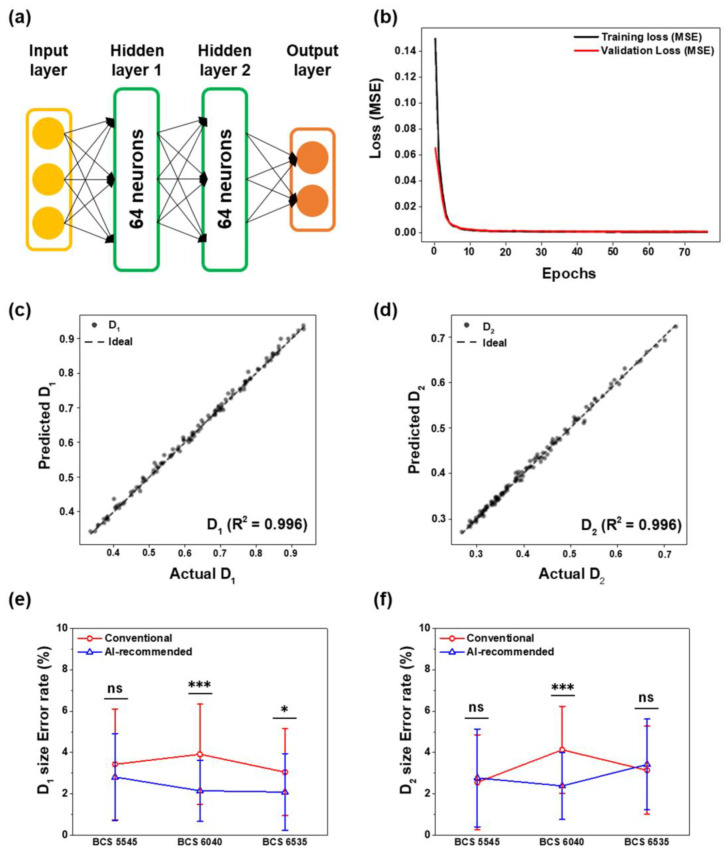
A multi-layer perceptron (MLP) model actual and predicted results: (**a**) architecture of the MLP model with two hidden layers, (**b**) training and validation loss curve, (**c**) comparison between predicted and actual values of D_1_, (**d**) comparison between predicted and actual values of D_2_, (**e**) comparison of D_1_ size error rates between experimental and AI-recommended printing conditions, (**f**) comparison of D_2_ size error rates between experimental and AI-recommended printing conditions. (*: *p* < 0.05, ***: *p* < 0.001, and ns (not significant): *p* ≥ 0.05).

To establish optimal printing conditions for the BCS scaffold, an AI-based approach was employed to recommend suitable process parameters. For model training, three input variables of pressure (180, 160, 140, 120 kPa), printing speed (40, 80, 120, 160 mm/min), and delay time (0.25, 0.5, 0.75, 1.0, 1.25, 1.5, 1.75 s) were systematically varied. For each parameter combination, the resulting D_1_ and D_2_ dimensions were measured and used as output variables to train the prediction model. The full dataset used for training is provided in Table S1, which includes measured values of D_1_ and D_2_ for all combinations of pressure, printing speed and delay time. All samples were printed under ambient conditions of 22 ± 3 °C and 47 ± 7% relative humidity. The overall architecture of the MLP-based artificial neural network used for this study is shown in Figure 2a. The network consists of an input layer, two hidden layers with 64 neurons each, and a two-node output layer representing D_1_ and D_2_. Figure 2b illustrates the training and validation loss (mean squared error, MSE) curves across epochs. Both losses rapidly decreased within the first 10 epochs and converged to near-zero values, indicating stable training convergence. The validation loss closely followed the training loss throughout, with no signs of divergence, suggesting strong generalization and minimal overfitting. Consistently, five-fold cross-validation yielded an average validation MSE of 0.000485 mm^2^, corresponding to an RMSE of approximately 0.022 mm. Given the target diameter range of 0.3–0.8 mm, the model achieved an RMSE of 0.022 mm (~22 μm), corresponding to 4% relative to the mean, indicating high predictive accuracy at the process scale. The prediction performance of the model is shown in Figure 2c,d, comparing the actual and predicted values of D_1_ and D_2_. The predicted values show a good agreement with the actual values, with coefficients of determination (R^2^) of 0.996 for both D_1_ and D_2_. The data points are tightly clustered along the ideal diagonal line, further confirming the high predictive performance of the model. These results demonstrate that the trained AI-based model effectively captures the relationships between printing process parameters (pressure, printing speed, delay) and strand geometry (D_1_ and D_2_). To further validate the model’s practical effectiveness, Figure 2e,f present a comparison of the dimensional error rates of D_1_ and D_2_ between samples fabricated using experimentally determined conditions and those produced using AI-recommended parameters. Among the experimental results, the conditions corresponding to “BCS 5545,” “BCS 6040,” and “BCS 6535,” which exhibited the lowest error rates, were selected for comparison. The parameter settings for these conditions are provided in Table 2. In both cases, the AI-recommended group consistently exhibited lower error rates, with statistically significant improvement observed in “BCS 6040”. These findings indicate that the proposed AI-assisted parameter selection framework helped enhance fabrication accuracy and consistency, supporting more reliable scaffold fabrication. Additionally, the specific printing conditions recommended by the AI-model are summarized in Table 3.

Previous studies have primarily applied AI in additive manufacturing for real time defect detection, or filament morphology prediction during extrusion, focusing on improving reliability and print quality [29,30]. In contrast, this study adopts a simpler yet effective approach by applying AI-based process optimization to adjust only key printing parameters (pressure, printing speed, and delay time) to achieve the desired BCS structure. This strategy enables accurate geometric reproduction with relatively straightforward experimental settings, offering a practical balance between precision and implementation simplicity. However, as the model is data-driven, its performance still relies on sufficient experimental data acquisition, which remains a limitation similar to conventional approaches.

### 3.2. Comparison of 3D Model and Scaffold for Morphological Fidelity

**Figure 3:**
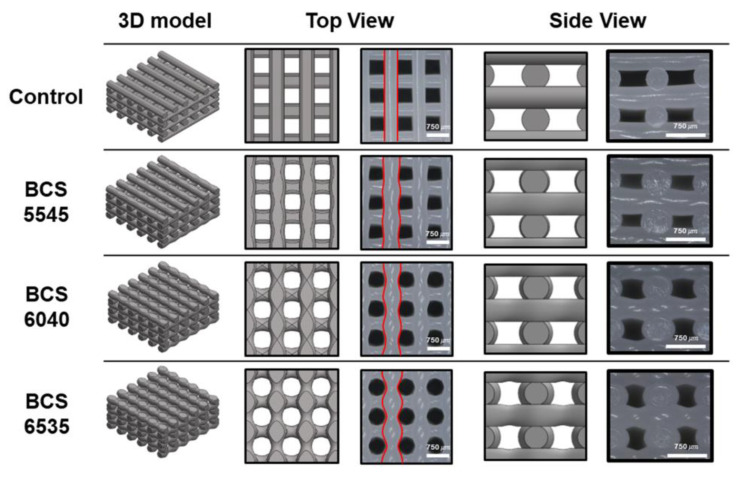
Three-dimensional medels and fabricated scaffold images for each scaffold type.

We successfully fabricated both the Control scaffold and BCS scaffolds using AM technology. The scaffolds were fabricated based on the optimal printing conditions derived from the AI based prediction model for the fabrication of D_1_ and D_2_. To evaluate the accuracy of the fabricated scaffolds, we compared the images of the designed scaffolds with those of the fabricated scaffolds. As shown in Figure 3, the fabricated scaffolds closely resembled the designed scaffolds, with no collapse or deformation of pores or strands.

**Figure 4:**
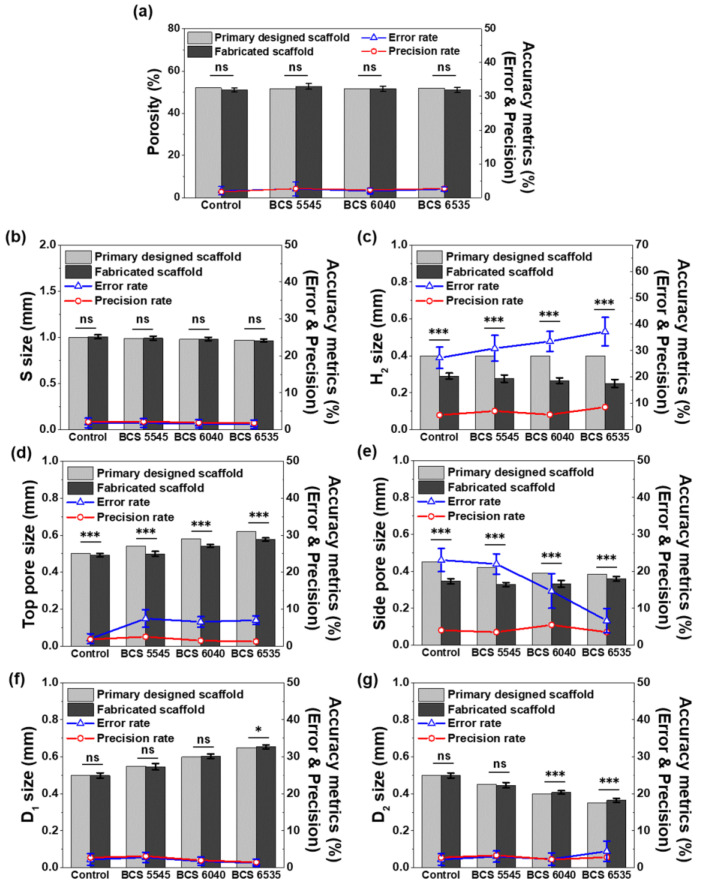
Morphological fidelity evaluation by comparing primary designed and measured values of each scaffold type: (**a**) porosity, (**b**) S size, (**c**) H_2_ size, (**d**) top pore size, (**e**) side pore size, (**f**) D_1_ size, and (**g**) D_2_ size. (*: *p* < 0.05, ***: *p* < 0.001, and ns (not significant): *p* ≥ 0.05).

To further assess the similarity, we compared their dimensional accuracy. Detailed quantitative data, including the target size, measured values, error rates, and precision rates for porosity, H_2_, S, D, D_1_, D_2_, top pore, and side pore size are systematically summarized in Tables S2–S9. As shown in Figure 4a,b there were no statistically significant differences in porosity and S size between the designed and fabricated scaffolds across all groups, indicating that this dimension was consistently well maintained throughout the fabrication process. In contrast, several other parameters specifically those shown in Figure 4d,f,g indicated significant differences between the designed scaffold and the fabricated scaffold. Despite these differences, the error-rate values (blue line) remained below 10%, indicating that the fabricated scaffold maintained a high level of accuracy compared to the designed target values. Additionally, the precision-rate value (red line) remained below 10%, indicating that the fabricated sample measurements were highly consistent with the mean fabricated value. This suggests that although differences from the designed values exist, the fabrication process itself was stable and highly reproducible. However, as shown in Figure 4c,e the error-rate value was relatively high, which can be attributed to the increased contact area resulting from the extended delay time. As the delay time increased, more material was extruded before movement continued, leading to an expanded contact area between strands. This in turn affected the H_2_ and side pore introducing increased variability. Since the total scaffold height was uniformly fixed at 2.4 mm, the increase in D_1_ value resulted in an expanded contact area, further amplifying the compression effect. As a result, the expanded contact area caused strand compression, leading to a reduction in H_2_ size in overlapping regions, which in turn resulted in a decrease in side pore size. Additionally, the material deposition process contributed to further strand height variation. With increased delay time, a larger volume of molten material was deposited before solidification, resulting in horizontal material spreading rather than maintaining the designed strand height (Figure S2). Therefore, H_2_ was further compressed in the overlapping regions, and the side pore was also affected, leading to a reduction in its size. As a result, both the increased contact area due to D_1_ and the material deposition influenced the final strand height variations, which in turn affected the side pore size.

### 3.3. Compressive Stiffness Comparison Between Primary Designed, Fabricated, and Secondary Re-Designed Scaffold

**Figure 5:**
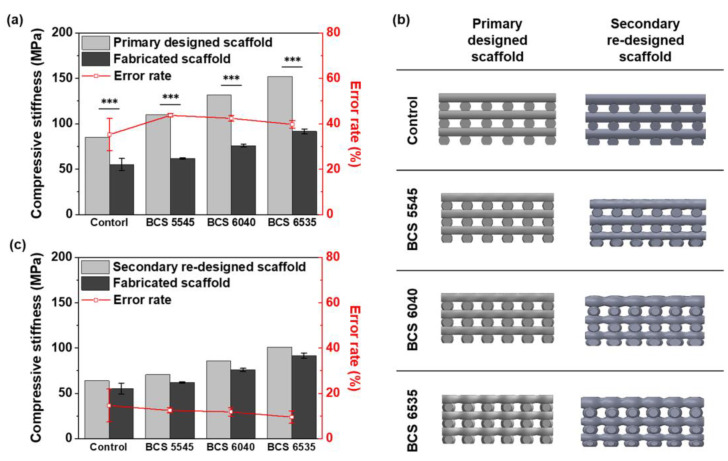
Compressive stiffness comparison between primary designed, fabricated, and secondary re-designed scaffolds: (**a**) compressive stiffness and error rate comparison graph between primary designed and fabricated scaffolds, (**b**) side view of 3D models of primary designed and secondary re-designed scaffold, (**c**) compressive stiffness and error rate comparison graph between secondary re-designed scaffold and fabricated scaffolds. (***: *p* < 0.001).

We conducted a simulation using ABAQUS to determine the compressive stiffness of the designed scaffolds. The effective stiffness (*E**^eff^***) was calculated using Equations (4)–(6).

(4) $$\sigma =E^{eff}\varepsilon$$

(5) $$E^{eff}=\frac{\sigma }{\varepsilon }$$

(6) $$E^{eff}=\frac{\frac{P}{A}}{\frac{\delta }{L}}$$

where σ is the stress and ε is the strain. The numerical compressive stiffness values obtained for the Control scaffold, “BCS 5545,” “BCS 6040,” and “BCS 6535” were 85.33 MPa, 109.92 MPa, 131.72 MPa, and 151.91 MPa, respectively. To validate these numerical results, we measured the compressive stiffness of the fabricated scaffolds using a universal mechanical testing machine (MTS E42, MTS Systems, Eden Prairie, MN, USA). For each scaffold type, five samples were measured. The experimental compressive stiffness values for the Control scaffold, “BCS 5545,” “BCS 6040,” and “BCS 6535” were recorded as 55.25 ± 6.06 MPa, 61.84 ± 0.84 MPa, 75.87 ± 1.65 MPa, and 91.57 ± 2.65 MPa, respectively. Compared to the Control scaffold, the compressive stiffness increased by approximately 11.92%, 37.31%, and 65.73% for “BCS 5545,” “BCS 6040,” and “BCS 6535”, respectively. As shown in the graph in Figure 5a, the experimental results displayed a trend similar to the numerical analysis, indicating that compressive stiffness increases with the expansion of strand-to-strand contact areas. However, we observed a significant discrepancy between the numerical and experimental results. To address this discrepancy, we performed secondary re-designed scaffolds of the fabricated scaffolds and recalculated their compressive stiffness through numerical simulation. The secondary re-designed scaffolds are shown in Figure 5b, with detailed views of the top and bottom layer shown in Figure S3. The numerical compressive stiffness values obtained from the secondary re-designed scaffolds were 63.85 MPa, 70.64 MPa, 86.01 MPa, and 101.17 MPa for “Control,” “BCS 5545,” “BCS 6040,” and “BCS 6535,”, respectively. As depicted in Figure 5a,c, the error rates in compressive stiffness between the primary designed scaffold and fabricated scaffold were 35.25%, 43.74%, 42.40% and 39.72% for “Control,” “BCS 5545,” “BCS 6040,” and “BCS 6535,”, respectively. In contrast, the error rates in compressive stiffness between the secondary re-designed scaffold and fabricated scaffold were reduced to 14.58%, 12.45%, 11.79%, and 9.48% for “Control,” “BCS 5545,” “BCS 6040,” and “BCS 6535,”, respectively. The error rates between the secondary re-designed scaffolds and the fabricated scaffolds were significantly reduced, indicating that the recalculated values closely matched the experimental results. Additionally, the remaining discrepancies, despite using secondary re-designed scaffolds, were attributed to the limitations of the secondary re-designed process itself. Specifically, the secondary re-designed scaffolds were generated by tracing the outer contour of the fabricated scaffolds from images, which introduced minor design errors. Therefore, we concluded that the residual differences were due to design to fabrication deviations inherent in the manufacturing process.

### 3.4. Comparison of Stress Distribution in Primary Designed and Secondary Re-Designed Scaffolds

**Figure 6:**
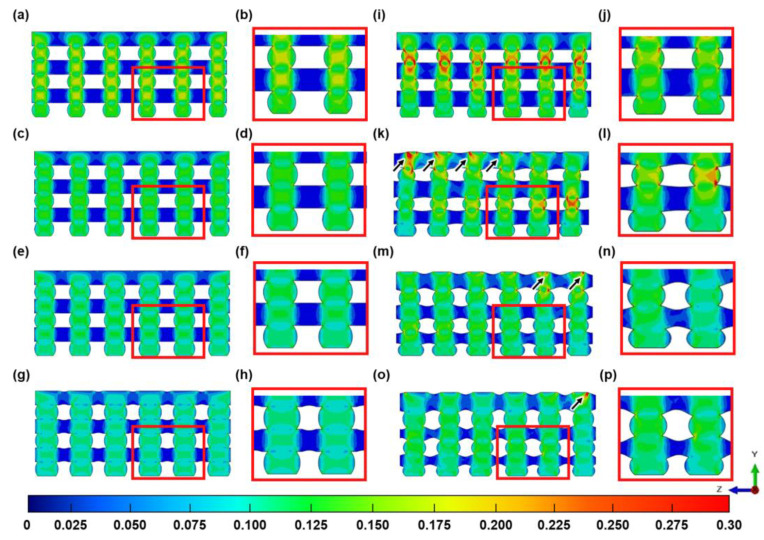
Side view images of the Von Mises stress on the scaffold: primary designed scaffold for (**a**) Control, (**c**) BCS 5545, (**e**) BCS 6040, (**g**) BCS 6535, enlarged stress views of primary designed scaffolds for (**b**) Control, (**d**) BCS 5545, (**f**) BCS 6040, (**h**) BCS 6535, secondary re-designed scaffold for (**i**) Control, (**k**) BCS 5545, (**m**) BCS 6040, (**o**) BCS 6535, enlarged stress views of secondary re-designed scaffolds for (**j**) Control, (**l**) BCS 5545, (**n**) BCS 6040, (**p**) BCS 6535.

As depicted in Figure 6d,f,h alongside Figure 6l,n,p it is evident that an increase in contact area leads to more effective dispersion of stress concentration compared to the Control (Figure 6b,j). The primary designed scaffold (Figure 6a,c,e,g) exhibits uniform stress distribution across all layers, whereas the secondary re-designed scaffold (Figure 6i,k,m,o) displays inconsistent stress distribution between layers. This difference arises from fabrication errors during the layer-by-layer printing process. Additionally, the bottom strand of the secondary re-designed scaffold shows noticeable spreading compared to the primary designed scaffold. The primary cause of this spreading is attributed to the delay time required to form D_1_, which extends the material solidification process. As a result, slower cooling and solidification lead to material spreading. Furthermore, during the initial stage of printing, the printer’s Y-axis starting position was set lower than the length of D_1_, contributing to the spreading phenomenon. Although increasing the Y-axis height during printing could diminish this spreading, it would introduce cumulative manufacturing errors as printing progresses. Moreover, in the overhanging strand region where delay time is not applied, the thickness of the strand becomes thinner depending on the feed rate, resulting in variations in the height of the topmost strand. As indicated by the black arrows in Figure 6k,m,o, non-negligible stress concentration is observed at the upper part of the scaffold. This is due to the upper surface being fabricated with an uneven, curved profile rather than a flat surface as intended in the design. Consequently, areas with relatively higher profiles cause localized stress concentrations. These results underscore that printing parameters, such as delay time and layer alignment, play a crucial role in determining the structural accuracy and mechanical behavior of the fabricated scaffold.

### 3.5. Comparison of Contact Area in Primary Designed and Fabricated Scaffold

**Figure 7:**
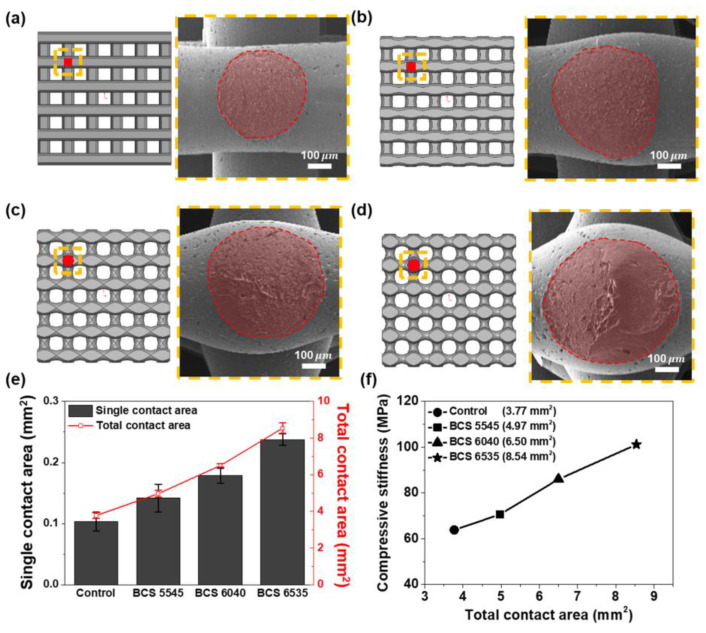
Comparison of interlayer strand contact area and compressive stiffness: interlayer contact area for (**a**) Control scaffold, (**b**) BCS 5545 scaffold, (**c**) BCS 6040 scaffold, (**d**) BCS 6535 scaffold, (**e**) graph of single contact area and total contact area, and (**f**) graph of compressive stiffness with respect to total contact area.

To verify whether compressive stiffness is correlated with contact area, we analyzed the contact areas using SEM images. As shown in Figure 7a–d, the SEM images clearly demonstrate a progressive increase in contact area among the scaffolds. Additionally, the graph in Figure 7e shows both the single and total areas measured for each group. The single contact areas for the Control scaffold, “BCS 5545,” “BCS 6040,” and “BCS 6535” which were 0.103 ± 0.015 mm^2^, 0.142 ± 0.022 mm^2^, 0.178 ± 0.012 mm^2^, and 0.238 ± 0.010 mm^2^ respectively. In the same manner, the total contact areas were 3.77 ± 0.141 mm^2^, 4.97 ± 0.181 mm^2^, 6.50 ± 0.100 mm^2^, and 8.54 ± 0.286 mm^2^. As shown in Figure 7f, compressive stiffness increases with the enlargement of the interlayer contact area. This result indicates that an increase in the contact area between strands in each layer leads to an increase in compressive stiffness.

### 3.6. Comparison of In Vitro Cell Proliferation Using the CCK-8 Assay

**Figure 8:**
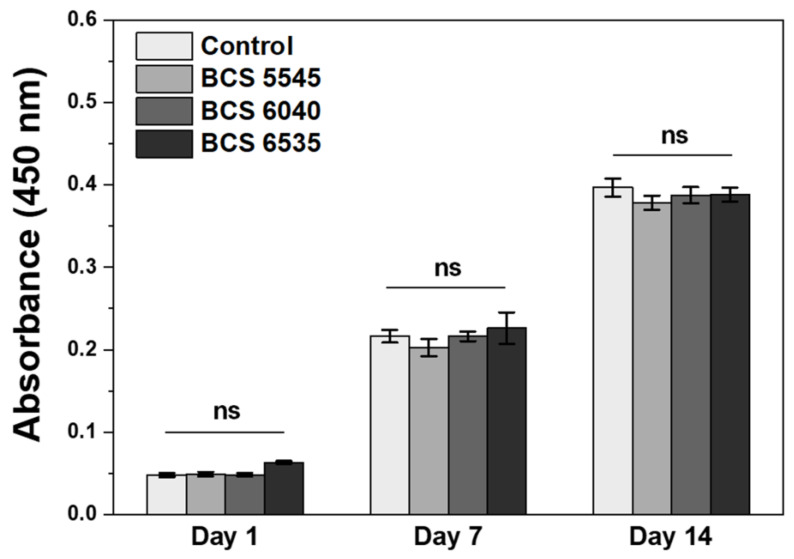
Cell proliferation evaluation for each scaffold type. (ns (not significant): *p* ≥ 0.05).

To evaluate the influence of scaffold structure on cellular behavior, we conducted a CCK-8 (Cell Counting Kit-8) assay, which measures the metabolic activity of viable cells to assess cell proliferation and viability. Scaffolds with sufficient porosity can provide a favorable environment for vascularization, nutrient and oxygen delivery, and waste removal [31,32]. The Control and BCS scaffolds were fabricated within the pore size range known to promote bone regeneration [33]. Additionally, all fabricated scaffolds were designed with a porosity of 50%. As shown in the graph in Figure 8, there were no significant differences in cell proliferation among the different scaffold types. This result indicates that the scaffold structure did not negatively affect cell proliferation. Therefore, we conclude that the designed scaffold structures provide a suitable environment for cell growth without inhibiting proliferation.

## 4. Conclusions

In this study, we proposed a BCS scaffold designed to increase the contact area between strands in each layer, thereby improving load transfer and compressive stiffness while maintaining the simplicity of grid structures. To further enhance fabrication efficiency and precision, an AI-based process optimization model was developed to determine optimal printing parameters (pressure, printing speed, and delay time) for accurate realization of the predesigned geometry. Using AM technology, we successfully fabricated the BCS scaffold through the printing process alone. Numerical analysis and UTM experiments confirmed that the BCS scaffold exhibited superior compressive stiffness compared to the Control scaffold. Additionally, through secondary redesigned, we identified discrepancies between the primary designed and fabricated scaffolds, highlighting primary design-to-fabrication errors. SEM images further confirmed an increase in contact area, and the results demonstrated that compressive stiffness increased proportionally with contact area. Finally, we evaluated cell proliferation through in vitro experiments using CCK-8 analysis. The results indicated that the scaffold structure did not inhibit cell proliferation. The novelty of this study lies in both eh introduction of a new BCS structural concept and the integration of an Ai-based process optimization framework to improve fabrication accuracy and reproducibility. Nonetheless, this study is limited to a single scaffold geometry (BCS structure) and a specific process parameter range. Future improvements may involve expanding the AI training dataset to include broader printing conditions and materials, thereby enhancing the model’s robustness and general applicability. In conclusion, applying an AI-based process optimization approach improved both fabrication efficiency and mechanical performance, demonstrating that the proposed BCS scaffold is a promising candidate for bone tissue regeneration applications.

## Figures and Tables

**Table 1:** Variables and porosity of the primary designed scaffold.

	H_1_ (mm)	H_2_ (mm)	L (mm)	W (mm)	D (mm)	D_1_ (mm)	D_2_ (mm)	S (mm)	Porosity (%)
Control	2.4	0.4	5.5	5.5	0.5			1.00	52.02
BCS 5545						0.55	0.45	0.99	51.72
BCS 6040						0.60	0.40	0.98	51.67
BCS 6535						0.65	0.35	0.97	51.91

**Table 2:** Conventionally selected printing conditions for BCS scaffolds and the measured D_1_ and D_2_ values.

	Pressure (kPa)	Printing Speed (mm/min)	Delay Time (s)	D_1_ Size (mm)	D_2_ Size (mm)
BCS 5545	140	40	0.25	0.538 ± 0.021	0.445 ± 0.015
BCS 6040	160	80	0.75	0.622 ± 0.017	0.413 ± 0.014
BCS 6535	140	160	1.5	0.667 ± 0.017	0.354 ± 0.013

**Table 3:** AI-recommended printing conditions for BCS scaffolds and the measured D_1_ and D_2_ values.

	Pressure (kPa)	Printing Speed (mm/min)	Delay Time (s)	D_1_ Size (mm)	D_2_ Size (mm)
BCS 5545	177	64.24	0.25	0.554 ± 0.019	0.443 ± 0.015
BCS 6040	179	107.88	0.71	0.596 ± 0.015	0.399 ± 0.012
BCS 6535	144	160	1.35	0.635 ± 0.018	0.343 ± 0.012

## Data Availability

The original contributions presented in this study are included in the article/Supplementary Material. Further inquiries can be directed to the corresponding author.

## Supplementary Materials

The following supporting information can be downloaded at: https://www.mdpi.com/article/10.3390/polym17222973/s1, Figure S1: Boundary conditions: (a) control scaffold and bead-chain-shaped scaffold boundary condition of calculating effective stiffness (b) control scaffold and bead-chain-shaped scaffold boundary condition of calculating von Mises stress, Figure S2: Comparison between the primary designed scaffold and the fabricated scaffold: (a) control (b) BCS 5545 (c) BCS 6040 (d) BCS 6535, Figure S3: Comparison between the primary designed scaffold and the secondary re-designed scaffold. (yellow boxes indicate the top layer, and red boxes indicate the bottom layer.); Table S1: Training dataset: measured scaffold diameters (D_1_, D_2_) under varying pressure, printing speed, and delay time, Table S2: Fabrication fidelity data of the scaffolds: Porosity, Table S3: Fabrication fidelity data of the scaffolds: H_2_ size, Table S4: Fabrication fidelity data of the scaffolds: S size, Table S5: Fabrication fidelity data of the scaffolds: D size, Table S6: Fabrication fidelity data of the scaffolds: D_1_ size, Table S7: Fabrication fidelity data of the scaffolds: D_2_ size, Table S8: Fabrication fidelity data of the scaffolds: Top pore size, Table S9: Fabrication fidelity data of the scaffolds: Side pore size.
