# Supplementary material for: Additive Manufacturing of Bead-Chain-Shaped Scaffolds with AI-Based Process Optimization
Source: Polymers (Basel). 2025 Nov 7;17(22):2973. doi: 10.3390/polym17222973 (PMC12656060; doi:10.3390/polym17222973)
Supplement: Supplementary file 1 [file polymers-17-02973-s001.zip › polymers-3878436-supplementary.pdf]

Supplementary material

AI-Optimized Design and Additive Manufacturing of Bead-Chain-Shaped Scaffolds: Fabrication and Characterization

*JinA Kim[1], Hyung Woo Kim[2, 3, 4, *], Young-Sam Cho[2, 3, 4, *]*

[1]Department of Mechanical Engineering, College of Engineering, Wonkwang University, 460 Iksandae-ro, Iksan Jeonbuk, 54538, Republic of Korea

[2]Division of Mechanical Engineering, College of Engineering, Wonkwang University, 460 Iksandae-ro, Iksan Jeonbuk, 54538, Republic of Korea

[3]MECHABIO Group, Wonkwang University, 460 Iksandae-ro, Iksan Jeonbuk, 54538, Republic of Korea

[4]Advanced Bio-Convergence Research Center, Wonkwang University, 460 Iksandae-ro, Iksan Jeonbuk, 54538, Republic of Korea

[*] Corresponding author:

- E-mail: kimhw1203@wku.ac.kr (H. W. Kim),
- E-mail: youngsamcho@wku.ac.kr (Y.-S. Cho),

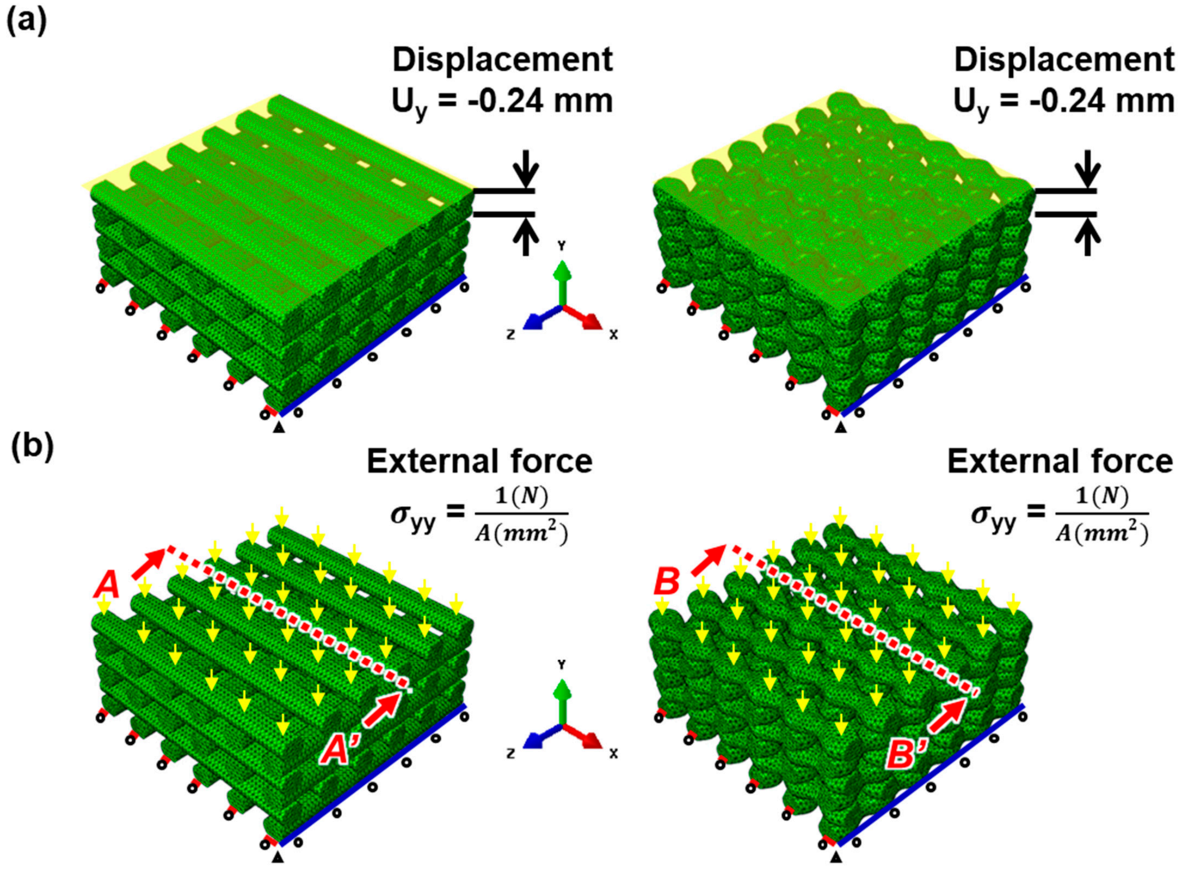

Figure S1. Boundary conditions: (a) control scaffold and bead-chain-shaped scaffold boundary condition of calculating effective stiffness (b) control scaffold and bead-chain-shaped scaffold boundary condition of calculating von Mises stress

Table S1. Training dataset: measured scaffold diameters (D_1, D_2) under varying pressure, printing speed, and delay time.

Pressure (kPa)	Printing speed (mm/min)	Delay time (s)	D_1 size (mm)	D_2 size (mm)
180	40	0.25	0.687 ± 0.023	0.553 ± 0.018
180	40	0.5	0.777 ± 0.022	0.611 ± 0.023
180	40	0.75	0.849 ± 0.026	0.663 ± 0.028
180	40	1.0	0.936 ± 0.030	0.725 ± 0.025
180	80	0.25	0.482 ± 0.019	0.390 ± 0.015
180	80	0.5	0.583 ± 0.022	0.437 ± 0.018
180	80	0.75	0.701 ± 0.023	0.470 ± 0.022
180	80	1.0	0.786 ± 0.025	0.512 ± 0.023
180	80	1.25	0.860 ± 0.023	0.569 ± 0.029
180	120	0.25	0.404 ± 0.015	0.342 ± 0.019
180	120	0.5	0.511 ± 0.022	0.356 ± 0.012
180	120	0.75	0.627 ± 0.021	0.382 ± 0.016
180	120	1.0	0.716 ± 0.021	0.414 ± 0.021
180	120	1.25	0.808 ± 0.017	0.472 ± 0.017
180	120	1.5	0.866 ± 0.023	0.508 ± 0.034
180	160	0.25	0.372 ± 0.017	0.313 ± 0.012
180	160	0.5	0.464 ± 0.021	0.318 ± 0.013
180	160	0.75	0.587 ± 0.019	0.334 ± 0.017
180	160	1.0	0.683 ± 0.020	0.365 ± 0.016
180	160	1.25	0.773 ± 0.020	0.406 ± 0.020
180	160	1.5	0.864 ± 0.019	0.468 ± 0.026
180	160	1.75	0.936 ± 0.027	0.530 ± 0.018
160	40	0.25	0.612 ± 0.020	0.494 ± 0.019
160	40	0.5	0.688 ± 0.019	0.541 ± 0.021
160	40	0.75	0.7871 ± 0.019	0.587 ± 0.019
160	40	1.0	0.847 ± 0.020	0.650 ± 0.026

**160**	**40**	**1.25**	**0.909 ± 0.023**	**0.702 ± 0.027**
**160**	**80**	**0.25**	**0.409 ± 0.016**	**0.379 ± 0.012**
**160**	**80**	**0.5**	**0.524 ± 0.017**	**0.396 ± 0.012**
**160**	**80**	**0.75**	**0.622 ± 0.017**	**0.413 ± 0.014**
**160**	**80**	**1.0**	**0.697 ± 0.016**	**0.451 ± 0.018**
**160**	**80**	**1.25**	**0.768 ± 0.017**	**0.489 ± 0.020**
**160**	**80**	**1.5**	**0.833 ± 0.020**	**0.534 ± 0.038**
**160**	**80**	**1.75**	**0.893 ± 0.022**	**0.604 ± 0.024**
**160**	**120**	**0.25**	**0.373 ± 0.018**	**0.316 ± 0.014**
**160**	**120**	**0.5**	**0.455 ± 0.018**	**0.332 ± 0.016**
**160**	**120**	**0.75**	**0.543 ± 0.020**	**0.341 ± 0.014**
**160**	**120**	**1.0**	**0.649 ± 0.013**	**0.383 ± 0.014**
**160**	**120**	**1.25**	**0.723 ± 0.016**	**0.427 ± 0.016**
**160**	**120**	**1.5**	**0.780 ± 0.019**	**0.462 ± 0.025**
**160**	**120**	**1.75**	**0.850 ± 0.018**	**0.529 ± 0.027**
**160**	**160**	**0.25**	**0.335 ± 0.012**	**0.289 ± 0.019**
**160**	**160**	**0.5**	**0.427 ± 0.016**	**0.299 ± 0.012**
**160**	**160**	**0.75**	**0.521 ± 0.017**	**0.313 ± 0.012**
**160**	**160**	**1.0**	**0.620 ± 0.017**	**0.339 ± 0.013**
**160**	**160**	**1.25**	**0.705 ± 0.018**	**0.364 ± 0.014**
**160**	**160**	**1.5**	**0.772 ± 0.018**	**0.408 ± 0.021**
**160**	**160**	**1.75**	**0.843 ± 0.025**	**0.458 ± 0.025**
**140**	**40**	**0.25**	**0.538 ± 0.021**	**0.445 ± 0.015**
**140**	**40**	**0.5**	**0.616 ± 0.021**	**0.478 ± 0.018**
**140**	**40**	**0.75**	**0.686 ± 0.022**	**0.511 ± 0.020**
**140**	**40**	**1.0**	**0.757 ± 0.021**	**0.552 ± 0.023**
**140**	**40**	**1.25**	**0.808 ± 0.021**	**0.594 ± 0.021**
**140**	**40**	**1.5**	**0.867 ± 0.022**	**0.644 ± 0.025**
**140**	**40**	**1.75**	**0.922 ± 0.029**	**0.684 ± 0.030**
**140**	**80**	**0.25**	**0.384 ± 0.013**	**0.346 ± 0.014**
**140**	**80**	**0.5**	**0.471 ± 0.017**	**0.356 ± 0.015**

**140**	**80**	**0.75**	**0.560 ± 0.020**	**0.369 ± 0.014**
**140**	**80**	**1.0**	**0.639 ± 0.017**	**0.397 ± 0.015**
**140**	**80**	**1.25**	**0.709 ± 0.021**	**0.432 ± 0.019**
**140**	**80**	**1.5**	**0.768 ± 0.020**	**0.461 ± 0.018**
**140**	**80**	**1.75**	**0.826 ± 0.019**	**0.508 ± 0.024**
**140**	**120**	**0.25**	**0.347 ± 0.018**	**0.315 ± 0.019**
**140**	**120**	**0.5**	**0.406 ± 0.014**	**0.307 ± 0.012**
**140**	**120**	**0.75**	**0.484 ± 0.017**	**0.323 ± 0.011**
**140**	**120**	**1.0**	**0.567 ± 0.015**	**0.341 ± 0.010**
**140**	**120**	**1.25**	**0.639 ± 0.018**	**0.363 ± 0.013**
**140**	**120**	**1.5**	**0.705 ± 0.020**	**0.389 ± 0.020**
**140**	**120**	**1.75**	**0.771 ± 0.020**	**0.430 ± 0.022**
**140**	**160**	**0.5**	**0.382 ± 0.019**	**0.283 ± 0.013**
**140**	**160**	**0.75**	**0.449 ± 0.016**	**0.298 ± 0.014**
**140**	**160**	**1.0**	**0.536 ± 0.016**	**0.311 ± 0.012**
**140**	**160**	**1.25**	**0.613 ± 0.019**	**0.339 ± 0.017**
**140**	**160**	**1.5**	**0.667 ± 0.017**	**0.354 ± 0.013**
**140**	**160**	**1.75**	**0.736 ± 0.021**	**0.391 ± 0.022**
**120**	**40**	**0.25**	**0.474 ± 0.020**	**0.421 ± 0.011**
**120**	**40**	**0.5**	**0.539 ± 0.019**	**0.439 ± 0.014**
**120**	**40**	**0.75**	**0.598 ± 0.018**	**0.462 ± 0.017**
**120**	**40**	**1.0**	**0.671 ± 0.019**	**0.493 ± 0.017**
**120**	**40**	**1.25**	**0.731 ± 0.017**	**0.518 ± 0.017**
**120**	**40**	**1.5**	**0.787 ± 0.019**	**0.546 ± 0.022**
**120**	**40**	**1.75**	**0.832 ± 0.018**	**0.585 ± 0.022**
**120**	**80**	**0.25**	**0.357 ± 0.011**	**0.320 ± 0.012**
**120**	**80**	**0.5**	**0.412 ± 0.015**	**0.332 ± 0.013**
**120**	**80**	**0.75**	**0.483 ± 0.016**	**0.345 ± 0.015**
**120**	**80**	**1.0**	**0.565 ± 0.019**	**0.366 ± 0.016**
**120**	**80**	**1.25**	**0.627 ± 0.014**	**0.684 ± 0.015**
**120**	**80**	**1.5**	**0.697 ± 0.22**	**0.415 ± 0.017**

**120**	**80**	**1.75**	**0.736 ± 0.015**	**0.442 ± 0.018**
**120**	**120**	**0.5**	**0.382 ± 0.015**	**0.286 ± 0.009**
**120**	**120**	**0.75**	**0.450 ± 0.015**	**0.307 ± 0.015**
**120**	**120**	**1.0**	**0.513 ± 0.014**	**0.319 ± 0.012**
**120**	**120**	**1.25**	**0.580 ± 0.013**	**0.331 ± 0.015**
**120**	**120**	**1.5**	**0.646 ± 0.016**	**0.360 ± 0.018**
**120**	**120**	**1.75**	**0.695 ± 0.018**	**0.385 ± 0.018**
**120**	**160**	**0.5**	**0.355 ± 0.012**	**0.268 ± 0.010**
**120**	**160**	**0.75**	**0.419 ± 0.017**	**0.286 ± 0.011**
**120**	**160**	**1.0**	**0.491 ± 0.015**	**0.290 ± 0.016**
**120**	**160**	**1.25**	**0.560 ± 0.018**	**0.306 ± 0.013**
**120**	**160**	**1.5**	**0.622 ± 0.016**	**0.321 ± 0.013**
**120**	**160**	**1.75**	**0.687 ± 0.015**	**0.355 ± 0.017**

Table S2. Fabrication fidelity data of the scaffolds: Porosity

	**Porosity target size (%)**	**Fabricated scaffold (%)**	**Error rate (%)**	**Precision rate (%)**
**Control**	**52.02**	**51.09 ± 0.88**	**2.05 ± 1.28**	**1.72**
**BCS 5545**	**51.72**	**52.76 ± 1.41**	**2.57 ± 2.06**	**2.68**
**BCS 6040**	**51.67**	**51.53 ± 1.18**	**1.89 ± 0.90**	**2.29**
**BCS 6535**	**51.91**	**51.05 ± 1.13**	**2.49 ± 0.69**	**2.22**

Table S3. Fabrication fidelity data of the scaffolds: H_2 size

	**H_2 target size (mm)**	**Fabricated scaffold (mm)**	**Error rate (%)**	**Precision rate (%)**
**Control**	**0.4**	**0.29 ± 0.016**	**27.26 ± 4.04**	**5.56**
**BCS 5545**		**0.28 ± 0.020**	**30.84 ± 4.90**	**7.08**
**BCS 6040**		**0.27 ± 0.015**	**33.48 ± 3.80**	**5.71**
**BCS 6535**		**0.25 ± 0.022**	**37.21 ± 5.38**	**8.56**

Table S4. Fabrication fidelity data of the scaffolds: S size

	**S target size (mm)**	**Fabricated scaffold (mm)**	**Error rate (%)**	**Precision rate (%)**
**Control**	**1.00**	**1.01 ± 0.022**	**1.84 ± 1.34**	**2.15**
**BCS 5545**	**0.99**	**0.99 ± 0.022**	**1.81 ± 1.20**	**2.19**
**BCS 6040**	**0.98**	**0.98 ± 0.019**	**1.59 ± 1.10**	**1.90**
**BCS 6535**	**0.97**	**0.97 ± 0.017**	**1.43 ± 1.13**	**1.81**

Table S5. Fabrication fidelity data of the scaffolds: D size

	D target size (mm)	Fabricated scaffold (mm)	Error rate (%)	Precision rate (%)
Control	0.5	0.50 ± 0.014	2.21 ± 1.56	2.74

Table S6. Fabrication fidelity data of the scaffolds: D_1 size

	D_1 target size (mm)	Fabricated scaffold (mm)	Error rate (%)	Precision rate (%)
BCS 5545	0.55	0.55 ± 0.017	2.77 ± 1.39	3.11
BCS 6040	0.60	0.60 ± 0.012	1.72 ± 1.12	1.96
BCS 6535	0.65	0.65 ± 0.010	1.29 ± 0.93	1.46

Table S7. Fabrication fidelity data of the scaffolds: D_2 size

	D_2 target size (mm)	Fabricated scaffold (mm)	Error rate (%)	Precision rate (%)
BCS 5545	0.45	0.45 ± 0.015	3.01 ± 1.52	3.35
BCS 6040	0.40	0.41 ± 0.010	2.31 ± 1.70	2.23
BCS 6535	0.35	0.37 ± 0.010	4.44 ± 2.75	2.81

Table S8. Fabrication fidelity data of the scaffolds: Top pore size

	Top pore target size (mm)	Fabricated scaffold (mm)	Error rate (%)	Precision rate (%)
Control	0.5	0.49 ± 0.009	2.03 ± 1.33	1.86
BCS 5545	0.54	0.50 ± 0.0013	7.48 ± 2.37	2.57
BCS 6040	0.58	0.54 ± 0.008	6.58 ± 1.41	1.51
BCS 6535	0.62	0.58 ± 0.007	7.02 ± 1.19	1.28

Table S9. Fabrication fidelity data of the scaffolds: Side pore size

	Side pore target size (mm)	Fabricated scaffold (mm)	Error rate (%)	Precision rate (%)
**Control**	**0.45**	**0.35 ± 0.014**	**23.04 ± 3.12**	**4.05**
**BCS 5545**	**0.42**	**0.33 ± 0.012**	**21.99 ± 2.75**	**3.53**
**BCS 6040**	**0.39**	**0.33 ± 0.018**	**14.66 ± 4.68**	**5.48**
**BCS 6535**	**0.385**	**0.36 ± 0.013**	**6.62 ± 3.30**	**3.53**

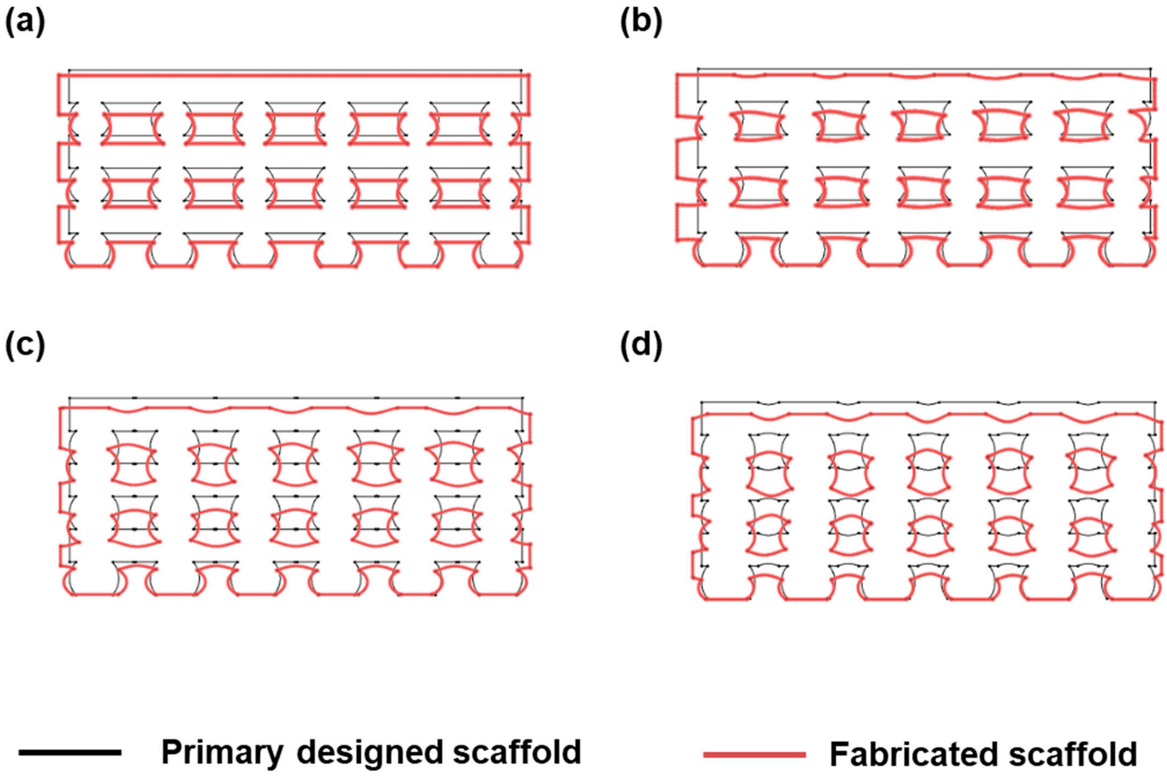

Figure S2. Comparison between the primary designed scaffold and the fabricated scaffold: (a) control (b) BCS 5545 (c) BCS 6040 (d) BCS 6535.

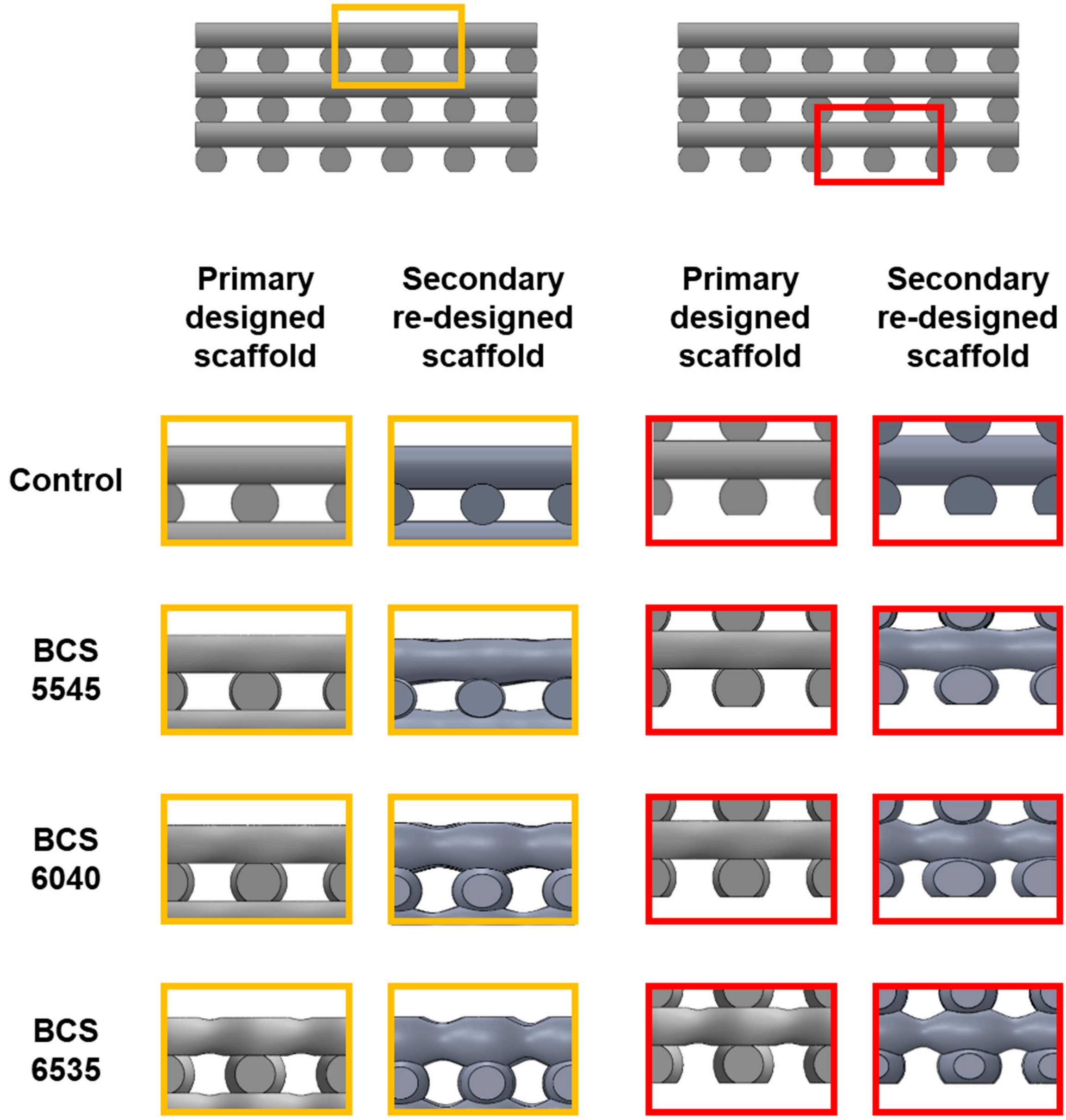

Figure S3. Comparison between the primary designed scaffold and the secondary re-designed scaffold. (yellow boxes indicate the top layer, and red boxes indicate the bottom layer.)
